# Diagnostik und Therapie bei Schwindel: Interdisziplinärer Leitfaden für die klinische Praxis

**DOI:** 10.1007/s00106-025-01598-0

**Published:** 2025-04-16

**Authors:** Alexander Andrea Tarnutzer, Hassen Kerkeni, Suzie Diener, Roger Kalla, Claudia Candreia, Renato Piantanida, Raphaël Maire, Antje Welge-Lüssen, Joris Budweg, Andreas Zwergal, Julia Dlugaiczyk

**Affiliations:** 1 Neurologie, Kantonsspital Baden, Baden, Schweiz; 2 Universität Zürich (UZH), Zürich, Schweiz; 3 Klinik für Neurologie, Inselspital Bern, Universität Bern, Bern, Schweiz; 4 Praxis „Neurologie St. Gallen“, St. Gallen, Schweiz; 5 Klinik für Hals‑, Nasen‑, Ohren- und Gesichtschirurgie, Kantonsspital Luzern, Luzern, Schweiz; 6 Servizio di Otorinolaringoiatria, Ente Ospedaliero Cantonale, Lugano, Schweiz; 7 Service d’oto-rhino-laryngologie, Centre hospitalier universitaire vaudois, Lausanne, Schweiz; 8 Hals-Nasen-Ohren-Klinik, Universitätsspital Basel, Basel, Schweiz; 9 Hausarztzentrum Bethesda, Basel, Schweiz; 10 Neurologische Klinik und Poliklinik, LMU Klinikum, München, Deutschland; 11 Deutsches Schwindel- und Gleichgewichtszentrum (DSGZ), LMU Klinikum, München, Deutschland; 12 Klinik für Ohren‑, Nasen‑, Hals- und Gesichtschirurgie, Universitätsspital Zürich (USZ), Rämistrasse 100, 8091 Zürich, Schweiz; 13 Interdisziplinäres Zentrum für Schwindel und neurologische Sehstörungen, Universitätsspital Zürich, Zürich, Schweiz

**Keywords:** Ataxie, HINTS, Lagerungsschwindel, Nystagmus, Vestibuläres Syndrom, Ataxia, HINTS, Positional vertigo, Nystagmus, Vestibular syndrome

## Abstract

**Hintergrund:**

Schwindel ist eines der häufigsten Leitsymptome in der klinischen Praxis, die Differenzialdiagnose stellt oftmals eine Herausforderung dar.

**Ziel der Arbeit:**

Die vorliegende Arbeit soll praxisorientierte und evidenzbasierte Empfehlungen zur Erstdiagnostik und -therapie von Schwindelsyndromen vermitteln.

**Material und Methoden:**

Der Konsensus einer interdisziplinären Arbeitsgruppe im Nachgang an eine Umfrage unter Schweizer Primärversorger:innen und Spezialist:innen (HNO, Neurologie) zu Diagnostik und Therapie bei Schwindel sowie die Ergebnisse einer Literaturrecherche in PubMed bis Oktober 2024 werden präsentiert.

**Ergebnisse und Schlussfolgerung:**

Eine strukturierte Anamnese und klinisch-neurootologische Untersuchung bilden die Grundlage für die Differenzialdiagnose der verschiedenen akuten (AVS) episodischen (EVS) und chronischen (CVS) vestibulären Syndrome (AVS: z. B. Schlaganfall oder akute unilaterale Vestibulopathie; EVS: z. B. benigner paroxysmaler Lagerungsschwindel [BPLS], M. Menière, vestibuläre Migräne, Vestibularisparoxysmie; CVS: z. B. bilaterale Vestibulopathie, „persistent postural perceptual dizziness“). Der vorliegende Artikel enthält Übersichten zu (i.) „red flags“ für eine potenziell gefährliche Ursache bei Patient:innen mit akutem Schwindel/Gangunsicherheit, (ii.) essenziellen klinisch-neurootologischen Untersuchungen bei Schwindel, (iii.) den Lagerungs- und Repositionsmanövern für BPLS der posterioren und lateralen Bogengänge und (iv.) den wichtigsten therapeutischen Ansätzen bei den o. g. vestibulären Syndromen sowie (v.) die „Top-10-Empfehlungen“ zu Anamnese, Diagnose und Therapie von Schwindelerkrankungen in der klinischen Praxis. Diese Zusammenstellung möge Ärzt:innen unterschiedlicher Disziplinen als tägliches „Vademecum“ bei der Erstdiagnose und -therapie von Schwindelerkrankungen dienen.

**Zusatzmaterial online:**

Die Online-Version dieses Beitrags (10.1007/s00106-025-01598-0) enthält Tab. S1 und Abb. S1–S2.

## Abstract (en)

**Background:**

Vertigo and dizziness belong to the most common leading symptoms in clinical practice. Their differential diagnosis, however, often poses a challenge.

**Objective:**

This work aims to provide evidence-based and practice-oriented recommendations for diagnosis and treatment of vertigo and dizziness in primary care.

**Materials and methods:**

The consensus statement of an interdisciplinary working group following a national survey among Swiss primary care physicians and neurotology specialists (neurologists, otorhinolaryngologists) is presented. The associated literature search in PubMed was conducted up to October 2024.

**Results and conclusion:**

Structured history taking and clinical neurotological examination form the basis for the differential diagnosis of the various acute (AVS), episodic (EVS), and chronic (CVS) vestibular syndromes (AVS: e.g., stroke or acute unilateral vestibulopathy; EVS: e.g., benign paroxysmal positional vertigo [BPPV], Menière’s disease, vestibular migraine, vestibular paroxysmia; CVS: e.g., bilateral vestibulopathy, persistent postural perceptual dizziness). The present paper covers the following topics: i) “red flags” for a potentially dangerous cause in patients with acute vertigo/dizziness/gait and balance disorders; ii) essential clinical neurotological examination steps; iii) diagnostic and therapeutic maneuvers for posterior and lateral canal BPPV; iv) the most important therapeutic strategies for the vestibular syndromes named above; and v) the top 10 recommendations regarding history taking, diagnosis, and treatment of vertigo and dizziness in clinical practice. This review aims to serve as a clinical companion for physicians of all specialties dealing with the primary diagnosis and treatment of vertigo and dizziness.

## Vorbemerkung

Im Nachgang an eine publizierte Umfrage unter Schweizer Primärversorger:innen und neurootologischen Spezialist:innen (HNO-Heilkunde, Neurologie) zum Thema Diagnostik und Therapie bei Schwindel wurde eine interdisziplinäre Arbeitsgruppe eingerichtet, bestehend aus Fachärzt:innen für Neurologie (Autoren AAT, HK, SD, RK), HNO-Heilkunde (CC, RP, RM, AWL, JD) und einem Allgemeinpraktiker (JB) aus der Schweiz sowie einem Vertreter aus Deutschland (AZ) [1–4]. Die vorliegende Arbeit stellt den Konsensus dieser Arbeitsgruppe dar und soll als Leitfaden für die Erstdiagnose und -therapie von Schwindelsyndromen in der klinischen Praxis verstanden werden.

## Häufigkeit

Schwank- oder Drehschwindel ist eines der häufigsten Leitsymptome und betrifft bis zu 8 % aller Arztkonsultationen [5]. Bei 10–15 % der Patient:innen besteht eine ernsthafte, aber behandelbare Erkrankung [6]. Eine ursächliche Verknüpfung zwischen bestimmten Schwindelqualitäten (Drehschwindel, „vertigo“; Schwank‑/Benommenheitsschwindel, „dizziness“; Präsynkope) und zugrunde liegenden Ursachen ist nicht zuverlässig möglich [7] und erlaubt keine Unterscheidung zwischen harmlosen und gefährlichen Erkrankungen [8]. Die breite Differenzialdiagnose, (zu) kurze Konsultationszeiten sowie mangelnde Expertise stellen wichtige limitierende Faktoren bei Primärversorger:innen dar [1].

Dieser Artikel setzt sich zum Ziel, bestehende Wissenslücken zu füllen sowie praxisorientierte und evidenzbasierte Empfehlungen zur Diagnostik und Therapie zu vermitteln.

## Diagnostik

### Anamnese

**Abb. 1:**
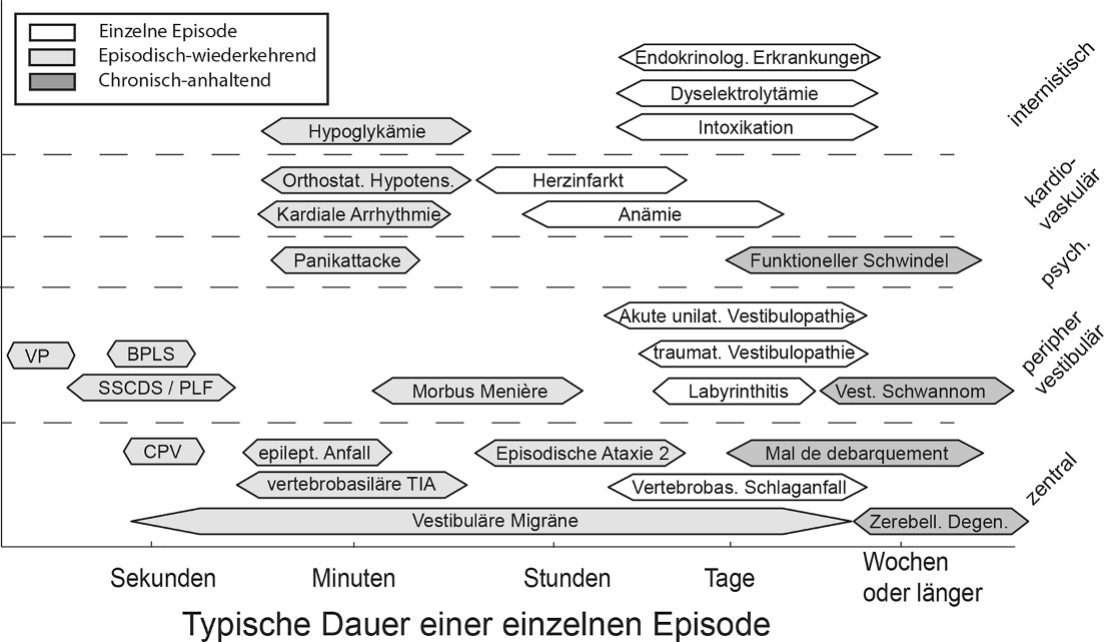
Die häufigsten Ursachen von Schwindel und deren Episodendauer. *BPLS* benigner paroxysmaler Lagerungsschwindel, *CPV* zentraler Lageschwindel, *PLF* Perilymphfistel, *SSCDS* Syndrom der superioren Bogengangdehiszenz, *TIA* transient-ischämische Attacke, *VP* vestibuläre Paroxysmie. (Mod. nach Büki und Tarnutzer [36], mit freundl. Genehmigung von Oxford Publishing Limited)

**Abb. 2:**
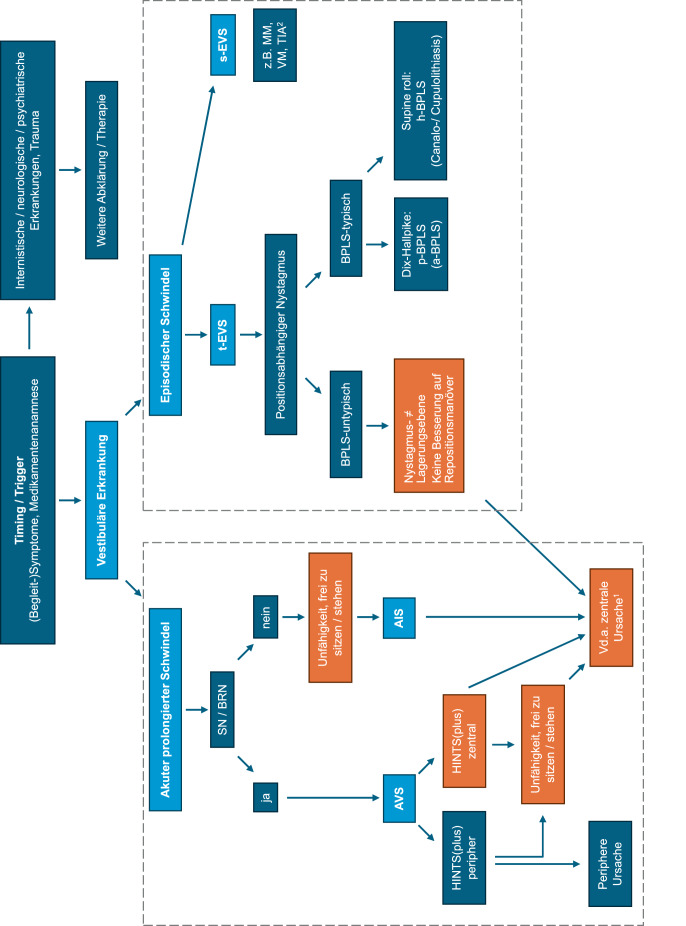
Differenzialdiagnose vestibulärer Störungen gemäß „TiTrATE“ („**Ti**ming, **Tr**iggers **A**nd **T**argeted **E**xaminations“). *Orange hinterlegt* „red flags“ für eine zentral-vestibuläre Störung. ^1^ Weitere „red flags“: vertikaler/torsioneller/vertikal-torsioneller Spontannystagmus, okuläre Lateropulsion, sakkadierte Blickfolge, dysmetrische Sakkaden (s. auch Tab. 1). ^2^ Die Unterscheidung zwischen einem akuten vestibulären Syndrom (*AVS*) und der Erstmanifestation eines spontanen episodischen vestibulären Syndroms (*s‑EVS*) ist oft erst retrospektiv möglich. Details siehe Haupttext. *a* anterior, *AIS* akutes Imbalance-Syndrom, *AVS* akutes vestibuläres Syndrom, *BPLS* benigner paroxysmaler Lagerungsschwindel, *BRN* Blickrichtungsnystagmus, *EVS* episodisches vestibuläres Syndrom, *h* horizontal, *HINTS* „head impulse, nystagmus, test of skew“, *MM* M. Menière, *p* posterior, *s* spontan, *t* getriggert, *SN* Spontannystagmus, *TIA* transient-ischämische Attacke, *VM* vestibuläre Migräne

Bei der Anamneseerhebung sollte ein Fokus auf die Dauer und Häufigkeit der Symptome (akut und prolongiert vs. episodisch vs. chronisch) sowie auf das Vorliegen von Triggern gelegt werden („Timing-and-Triggers-Herangehensweise“ [7]). Während wiederkehrender Schwindel (episodisches vestibuläres Syndrom, EVS) über Minuten bis Stunden im Rahmen einer vestibulären Migräne oder eines M. Menière auftreten kann, ist bei einer Erstepisode z. B. auch eine transient-ischämische Attacke (TIA) denkbar. Bei akutem prolongiertem Schwindel (akutes vestibuläres Syndrom, AVS) ist die Abgrenzung zwischen gefährlichen zentralen (z. B. Schlaganfall) und peripheren (z. B. akute unilaterale Vestibulopathie, AUVP) Ursachen essenziell (Abb. 1). Wichtig ist zudem die Unterscheidung zwischen Provokationsfaktoren (lösen Symptomatik aus) und exazerbierenden Faktoren (verstärken vorbestehende Symptomatik). Begleitsymptome, Vorerkrankungen und die aktuelle Medikation bilden weitere Themen. Die Abb. 2 skizziert das differenzialdiagnostische Vorgehen bei AVS und EVS gemäß „TiTrATE“ („**Ti**ming, **Tr**iggers **A**nd **T**argeted **E**xaminations“). Die wichtigsten „red flags“ finden sich in Tab. 1, essenzielle neurootologische Fachbegriffe sind im Glossar (Supplement, Tab. S1) aufgeführt.

**Tab. 1:** „Red flags“ bei Patient:innen mit akutem Schwindel und/oder Gangunsicherheit

„Red flag“ – Schwindel/Gangunsicherheit plus	Differenzialdiagnose	Empfohlene Maßnahme(n)
*Anamnese*		
Akute Hörminderung ohne Hinweise für eine Ohrerkrankung inkl. M. Menière (siehe auch Haupttext)	Zentrale Ursache (meist Hirnstamminfarkt im AICA-Stromgebiet)	Zuweisung auf die Stroke Unit zur weiteren Diagnostik/Therapie einschließlich MRT des Neurokraniums
Multiple vaskuläre Risikofaktoren (ABCD2-Score $\geq 4$)	Zentrale, meist ischämische Ursache	Zuweisung auf die Stroke Unit zur weiteren Diagnostik/Therapie einschließlich MRT des Neurokraniums
Perakuter Beginn ohne Trigger	Zentrale, meist ischämische Ursache	Zuweisung auf die Stroke Unit zur weiteren Diagnostik/Therapie einschließlich MRT des Neurokraniums
Neuartiger Kopf- oder Nackenschmerz ± neuartige einseitige Hörminderung	Erhöhter intrakranieller Druck (z. B. durch eine Raumforderung in der hinteren Schädelgrube oder eine Sinusvenenthrombose), Dissektion A. vertebralis	Zuweisung in die Notfallabteilung zur zerebralen Bildgebung (CT inkl. i.v. Kontrastmittel und Darstellung der Sinusvenen sowie der arteriellen hirnversorgenden Gefäße)
Bewusstseinsverlust	Epileptischer Anfall, erhöhter intrakranieller Druck mit drohender Herniation, Subarachnoidalblutung, Medikamentenintoxikation (z. B. Neuroleptika, Antiepileptika), kardiale Arrhythmie, Basilaristhrombose	Zuweisung in die Notfallabteilung zur zerebralen Bildgebung (CT inkl. CT-Angiographie ausreichend in den meisten Fällen), EEG, EKG, Medikamentenspiegelbestimmung
Anhaltendes Erbrechen, verminderte Nahrungsaufnahme oder Malabsorption	Akuter Thiaminmangel	Thiaminspiegelbestimmung im Blut, umgehende hochdosierte Thiaminsupplementation (3 × 200–500 mg pro Tag als Kurzinfusion über 5 Tage)
Fieber	Meningitis, Meningoenzephalitis	Zuweisung in die Notfallabteilung zur zerebralen Bildgebung (CT i. d. R. ausreichend) sowie zur Lumbalpunktion
Diplopie, Dysphagie, Dysarthrie, Dysästhesie, Dysmetrie („deadly Ds“)	Zentrale Ursache im Hirnstamm/Kleinhirn (Schlaganfall, Blutung, Tumor, demyelinisierende Läsion), Medikamentenintoxikation	Zuweisung auf die Stroke Unit zur weiteren Diagnostik/Therapie einschließlich MRT des Neurokraniums
*Klinische Befunde am Patientenbett*		
Normaler Kopfimpulstest (trotz Vorliegen eines Spontannystagmus oder Blickrichtungsnystagmus)	Zentrale Ursache im Hirnstamm/Kleinhirn (Schlaganfall, Blutung, demyelinisierende Läsion), Medikamentenintoxikation, Erstmanifestation einer vestibulären Migräne	Zuweisung auf die Stroke Unit zur weiteren Diagnostik/Therapie einschließlich MRT des Neurokraniums
Rein vertikaler, rein torsioneller oder torsionell-vertikaler Spontannystagmus	Zentrale Ursache im Hirnstamm/Kleinhirn (Schlaganfall, Blutung, demyelinisierende Läsion), Medikamentenintoxikation, akuter Thiaminmangel, Erstmanifestation einer vestibulären Migräne	Siehe oben
Horizontaler, nicht erschöpflicher Blickrichtungsnystagmus bei exzentrischer Blickwendung	Zentrale Ursache im Hirnstamm oder Kleinhirn (Schlaganfall, Blutung, demyelinisierende Läsion, Tumor), Erstmanifestation einer vestibulären Migräne, Medikamentenintoxikation, Thiaminmangel	Siehe oben
Klinisch manifeste vertikale Divergenz („skew deviation“) im alternierenden Abdecktest	Zentrale Ursache im Hirnstamm oder Kleinhirn (Schlaganfall, Blutung, demyelinisierende Läsion)	Siehe oben
(Neuartige) Okulomotorikstörung (sakkadierte Blickfolge, dysmetrische Sakkaden, Reboundnystagmus, vertikaler Nystagmus nach Kopfschütteln in der horizontalen Ebene)	Zentrale Ursache im Hirnstamm oder Kleinhirn (Schlaganfall, Blutung, demyelinisierende Läsion, neurodegenerativ, Tumor)	Bei akuter Symptomatik Vorstellung in der Notfallabteilung (inkl. zerebraler Bildgebung), bei subakuter/chronischer Symptomatik Zuweisung zur fachärztlich neurologischen Abklärung
Akuter einseitiger Hörverlust ohne Hinweise für eine Ohrerkrankung inkl. M. Menière	Zentrale Ursache (meist Hirnstamminfarkt im AICA-Stromgebiet)	Zuweisung auf die Stroke Unit zur weiteren Diagnostik/Therapie einschließlich MRT des Neurokraniums
Schwere Rumpfataxie (Unfähigkeit, ohne Hilfe/ohne Abstützen zu sitzen/stehen)	Vertebrobasiläre Ischämie, Hirnstamm‑/Kleinhirnblutung, demyelinisierende Läsion	Siehe oben
Positionsabhängiger Nystagmus ohne Besserung nach wiederholten Repositionsmanövern und/oder atypischer Präsentation (Schlagrichtung nicht passend zu einem Bogengang)	Zentrale Ursache im Hirnstamm oder Kleinhirn (Schlaganfall, Blutung, demyelinisierende Läsion, Tumor), Erstmanifestation einer vestibulären Migräne	Siehe oben
Auffällige Vitalzeichen (Hypotonie, Tachykardie, Arrhythmie)	Internistisch-kardiologische Ursachen	Dringliche kardiologische/internistische Abklärung

*AICA* anteriore inferiore zerebelläre Arterie, *CT* Computertomographie, *EEG* Elektroenzephalographie, *MRT* Magnetresonanztomographie

#### Empfehlung 1.

Priorität sollte die Suche nach gefährlichen akuten Schwindelursachen haben und dabei auf neurologische (z. B. Doppelbilder, [Hemi-]ataxie, Paresen, Dysarthrie), otologische (Ohrenschmerzen, Hörminderung) und kardiovaskuläre Begleitsymptome (Palpitationen, Arrhythmien, retrosternaler Druck) geachtet werden [7].

#### Empfehlung 2.

Bei der Anamneseerhebung sollte immer nach dem „Timing“ (Häufigkeit, Akuität, Dauer einzelner Episoden), Triggern (Provokations‑/Exazerbationsfaktoren), Begleitsymptomen sowie der aktuellen Medikation gefragt werden.

#### Empfehlung 3.

Die Qualität der Schwindelbeschwerden darf nicht zum Ausschluss einzelner Differenzialdiagnosen führen.

### Klinische Untersuchung bei akutem Schwindel

Essenziell ist die Unterscheidung zwischen benignen und gefährlichen Krankheitsbildern [9]. Besteht ein akuter prolongierter Schwindel mit Nausea/Vomitus, Gangunsicherheit, Bewegungsintoleranz sowie Nystagmus, so wird von einem AVS gesprochen. Liegt leitsymptomatisch eine Stand- und Gangunsicherheit ohne Nystagmus vor, wird dies als akutes Imbalance-Syndrom (AIS) bezeichnet (Tab. S1, Abb. 2).

Dabei ist die Suche nach subtilen okulomotorischen Zeichen sowie nach Stand- und Gangstörungen essenziell. Hierzu zählen zentrale Augenbewegungsstörungen ohne eingeschränkte Augenmotilität, insbesondere ein vertikaler, torsioneller oder vertikal-torsioneller Spontannystagmus (SN), ein horizontaler Blickrichtungsnystagmus (BRN), eine okuläre Lateropulsion, sakkadierte Folgebewegungen, dysmetrische Sakkaden und ein intakter vestibulookulärer Reflex (Tab. 1; Tab. S1; [10]). Offensichtliche fokal-neurologische Befunde fehlen bei gut der Hälfte der Patient:innen mit zentralem AVS. Die klinische Untersuchung ist hier sensitiver als eine frühe (d. h. innerhalb von 24–48 h nach Symptombeginn durchgeführte) Magnetresonanztomographie (MRT) des Neurokraniums mit diffusionsgewichteten Sequenzen (ca. 20 % falsch-negative Befunde) [10]. Das Vorliegen eines Spontannystagmus weist auf eine akute vestibuläre Störung (zentral oder peripher) hin. Sofern ein (isoliert) vertikaler, ein isoliert torsioneller oder torsionell-vertikaler SN vorliegt, spricht dies für eine zentrale Ursache (Tab. 1 und 2). Ein horizontal-torsioneller SN lässt keine Unterscheidung peripher vs. zentral zu [11].

**Tab. 2:** Essenzielle klinisch-neurologische Untersuchungen bei Schwindel

Untersuchung	Wann indiziert?	Interpretation eines pathologischen Befundes
Suche nach fokal-neurologischen Defiziten	Bei allen Patient:innen	Vorliegen fokal-neurologischer Defizite → starker Hinweis für eine zentrale Ursache
Suche nach einem Spontannystagmus mit/ohne Fixation (Frenzel-Brille)	Bei allen Patient:innen	Sofern horizontal, mit Torsion und/oder Abnahme bei Fixation → eher peripher
		Sofern rein vertikal, rein torsionell oder vertikal-torsionell und ohne Abnahme bei Fixation → eher zentral
Suche nach subtilen okulomotorischen (und audiologischen) Zeichen (HINTS plus)	Bei akutem prolongiertem Schwindel (AVS)	Kopfimpulstest → Einstellsakkade meist hinweisend auf periphere Ursache (*Cave:* „falsch“ abnorm bei 20 % der Patient:innen mit zentralem AVS), intakter Kopfimpulstest im Kontext eines AVS → suggestiv für zentrale Ursache
		Exzentrische Blickhaltefunktion → Blickrichtungsnystagmus (d. h. Drift der Augen in die Primärposition und Korrektursakkade nach außen) suggestiv für zentrale Ursache
		Alternierender Abdecktest → klare vertikale Einstellbewegung („skew deviation“) suggestiv für zentrale Ursache
		Neuartiger einseitiger Hörverlust (Prüfung mittels Flüsterzahlen oder Fingerreiben) ohne Hinweis für Ohrerkrankung → suggestiv für zentrale Ursache („Plus-Kriterium“)
Suche nach einer Rumpfataxie	Bei allen Patient:innen	Freies Sitzen oder Stehen ohne Abstützen nicht möglich → zentral
		Freies Stehen (im Tandemstand) und freies Gehen möglich → peripher oder zentral-vestibulär
Provokationsmanöver für die posterioren und lateralen Bogengänge	Bei typischen Provokationsfaktoren	Hallpike-Dix-Manöver: torsionell-geotroper Nystagmus → BPLS des posterioren Bogengangs
		Supine-Roll-Manöver: geotroper oder apogeotroper rein horizontaler Nystagmus → BPLS des lateralen Bogengangs

*AVS* akutes vestibuläres Syndrom, *BPLS* benigner paroxysmaler Lagerungsschwindel, *HINTS* „head impulse, nystagmus, test of skew“ (Kopfimpulstest, exzentrische Blickhaltefunktion, alternierender Abdecktest)

### Prüfung von HINTS sowie der Stand- und Gangfunktion

Liegt bei Patient:innen mit akutem prolongiertem Schwindel i.S.e. AVS ein Spontan- oder Blickrichtungsnystagmus vor, so ist die Prüfung von HINTS (**h**ead **i**mpulse, **n**ystagmus, **t**est of **s**kew) essenziell (Abb. 2, Tab. 2, Tab. S1). Eine zuverlässige Durchführung (Dauer 2–3 min) und Interpretation der HINTS-Untersuchung ist bereits nach wenigen Stunden Training möglich [12] und erlaubt die Identifikation zentraler Ursachen mit hoher Genauigkeit (Sensitivität 95,3 % [10]). Dabei sollte zwischen „peripheren HINTS“ und „zentralen HINTS“ unterschieden werden. Der horizontale Kopfimpulstest („**h**ead **i**mpulse“, KIT) dient der Prüfung des vestibulookulären Reflexes (VOR) der horizontalen Bogengänge (Tab. S1). Es sollten rasche, kleinamplitudige (5–15° Auslenkung) Kopfrotationen in nicht vorhersehbarer Richtung und Amplitude appliziert werden, während die Patient:innen die Nasenspitze des Untersuchenden fixieren [13]. Das Vorliegen von Korrektursakkaden spricht für eine VOR-Unterfunktion auf derjenigen Seite, zu welcher die rasche Kopfdrehung erfolgt. Dieser Befund ist obligat für die AUVP, findet sich aber auch bei ca. 20 % aller zentralen Ursachen (zweites Neuron des VOR liegt im Vestibulariskern im Hirnstamm). Daher zeigt nur ein unauffälliger KIT in Kombination mit einem SN klar eine zentrale Schwindelursache an. Weitere okulomotorische Zeichen wie die exzentrische Blickhaltefunktion („**n**ystagmus“: Prüfung bei 30° Lateralblick, Dauer $\geq$ 10 s) sowie der alternierende Abdecktest („**t**est of **s**kew“: abwechselndes Abdecken eines Auges, Frequenz = 0,5 Hz) sollten immer ergänzend geprüft werden (Tab. 2; Abb. 2).

Als „zentral“ gelten die HINTS-Befunde, sofern eines/mehrere der 3 Zeichen auf eine zentrale Ursache hinweisen (Tab. 2, Tab S1). Sind die HINTS-Befunde „peripher“, so sollte zusätzlich nach einer neu aufgetretenen einseitigen Hörminderung gesucht werden, was als „HINTS plus“ bezeichnet wird ([12]; Tab. 2; Tab. S1; Abb. 2). Liegt eine solche vor, spricht dies bis zum Beweis des Gegenteils (Innenohrerkrankung wie z. B. Erstmanifestation eines M. Menière) für eine zentrale Ursache. Bei Patient:innen mit AVS sollte daher immer auch eine Otoskopie durchgeführt werden (s. unten).

Ergänzend (oder alternativ, sofern keine Erfahrung mit HINTS besteht) sollte eine graduierte Prüfung der posturalen Stabilität und des Gangbilds erfolgen (Tab. 2; Abb. 2). Dies insbesondere, sofern kein Spontan- oder Blickrichtungsnystagmus vorliegt, aber ein anhaltender Schwindel und eine Gangunsicherheit bestehen. Dann ist von einem AIS auszugehen. Ist das selbständige Stehen/Sitzen nicht mehr möglich, so ist dies ein deutlicher Hinweis für eine zentrale Ursache [12].

Aufgrund akuter Behandlungsmöglichkeiten bei zentralen (meist ischämischen) Ursachen eines AVS oder AIS einschließlich einer intravenösen Thrombolyse (bis maximal 9 h nach Symptombeginn in ausgewählten Fällen möglich [14]) und einer endovaskulären Thrombektomie (bis maximal 24 h nach Symptombeginn in ausgewählten Fällen möglich [15]) sollten entsprechende Abklärungen umgehend und in einem Krankenhaus mit einer Stroke Unit erfolgen.

### Klinische Untersuchung bei spontanem episodischem oder chronischem Schwindel

Primäres Ziel ist die Unterscheidung zwischen peripher-, zentral- und nichtvestibulären Ursachen (Abb. 1). Okulomotorische Zeichen (z. B. sakkadierte Blickfolge, dysmetrische Sakkaden, vertikaler SN) sprechen für eine zentrale Ursache (Tab. 1 und 2), ein bilateral abnormer KIT in Kombination mit einem deutlichen Schwanken im Romberg-Test ist suggestiv für eine bilaterale Vestibulopathie (BVP). Bei Schwindel im Stehen/Gehen respektive einer chronischen Gangstörung sollte ein erweitertes Spektrum (inkl. Polyneuropathie [PNP] und zerebellären/hypokinetisch-rigiden Erkrankungen) berücksichtigt werden.

### Lagerungsmanöver

**Abb. 3:**
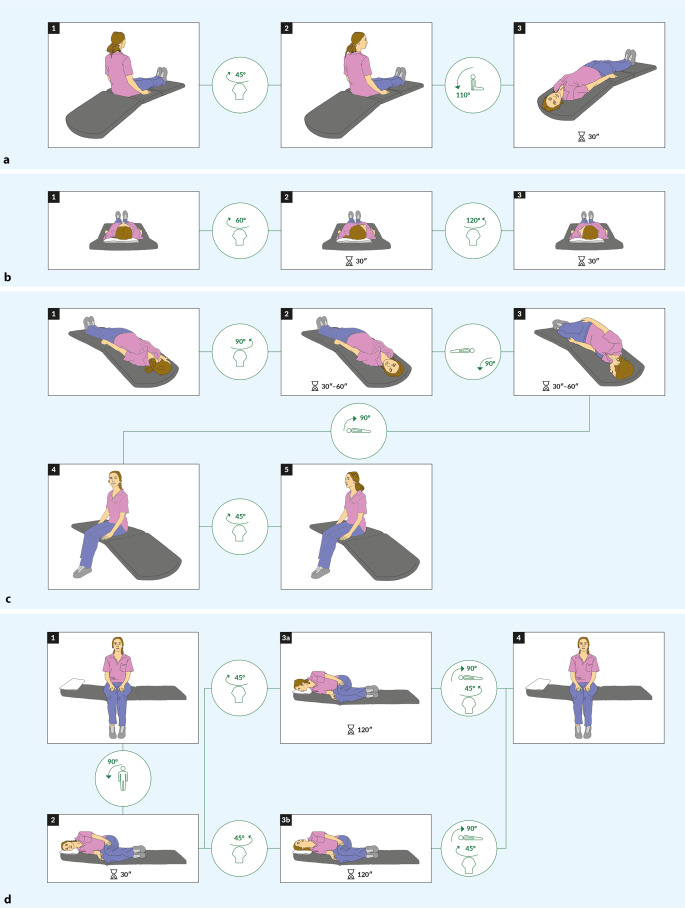
Lagerungs- und Repositionsmanöver beim benignen paroxysmalen Lagerungsschwindel (*BPLS*), schrittweise dargestellt und mit Piktogrammen zur Illustration der Kopf- und Körperpositionsänderungen (dabei immer Betrachtung des Patienten/der Patientin von hinten). **a** Mittels Hallpike-Dix-Manöver erfolgt die Prüfung der posterioren Bogengänge (hier: rechter posteriorer BG). Dabei wird der Patient/die Patientin mit einer Kopfrotation von ca. 45° zur Seite in eine Kopftieflage (ca. 20° unterhalb der Horizontalen) gebracht. Ein Nachweis eines torsionell-geotropen (d. h. drehend und zur Erde hin schlagenden) Nystagmus sowie Schwindel ist suggestiv für einen BPLS. **b** Die Prüfung der lateralen Bogengänge erfolgt mittels Supine-Roll-Test. Dabei wird in Rückenlage – oder mit leicht (10–20°) angehobenem Kopfteil – eine Kopfdrehung zu beiden Seiten von je ca. 60° durchgeführt und auf das Auftreten eines horizontalen geotropen oder apogeotropen (d. h. von der Erde weg schlagenden) Nystagmus geachtet (siehe Haupttext zur Bestimmung der betroffenen Seite). **c** Die Reposition eines BPLS des rechten posterioren Bogengangs mittels Epley-Manöver erfolgt schrittweise wie abgebildet und mit ausreichenden Pausen (30–60 s) pro Position. **d** Bei Nachweis eines BPLS des lateralen Bogengangs ist für die Reposition mittels Gufoni-Manöver die Unterscheidung zwischen der geotropen Variante (*3a*, hier für den linken lateralen Bogengang → es erfolgt eine Kopfrotation „nose down“) und der apogeotropen Variante (*3b*, hier für den rechten lateralen Bogengang → es erfolgt eine Kopfrotation „nose up“) entscheidend

Besteht ein episodischer *getriggerter* Schwindel, sollten die HINTS(plus)-Tests nicht primär durchgeführt werden, sondern die Suche nach einem benignen paroxysmalen Lagerungsschwindel (BPLS) mittels Lagerungsmanövern priorisiert werden ([16]; Tab. 2; Abb. 2). Aufgrund der Häufigkeit eines BPLS sollten Lagerungsmanöver immer bei einer klinischen Schwindelabklärung erfolgen. Während das Hallpike-Dix-Manöver (Abb. 3a) der Prüfung der posterioren Bogengänge (BG) dient, lässt sich mittels Supine-Roll-Manöver (Abb. 3b) ein BPLS des lateralen BG nachweisen (Tab. 2). Der Nachweis eines Crescendo-decrescendo-Nystagmus mit torsionell-geotroper Schlagrichtung (d. h. rotatorisch sowie zur Erde schlagend) sowie die Angabe von transientem Schwindel in der Hallpike-Dix-Position sprechen für einen BPLS des posterioren BG. Charakteristisch sind eine Latenz von wenigen Sekunden und eine Nystagmus- und Symptomdauer von 10–20 s („Canalolithiasis“) [16]. Ein horizontaler geotroper (zur Erde schlagender) oder apogeotroper (von der Erde weg schlagender) Nystagmus begleitet von Schwindel im Supine-Roll-Manöver spricht für einen BPLS des lateralen BG. Bei der geotropen Variante ist die betroffene Seite diejenige mit mehr Nystagmus, bei der apogeotropen Variante ist es diejenige Seite mit weniger Nystagmus. Dabei ist das Vorliegen eines geotropen (oder apogeotropen) Nystagmus sowohl bei Kopfdrehung nach links wie auch nach rechts erforderlich. Eine CT- oder MRT-Bildgebung des Neurokraniums bei Verdacht auf BPLS ist primär nicht nötig [17]. Eine MRT ist in therapierefraktären/atypischen Fällen zu erwägen (Tab. 1; Abb. 2). Im Fall eines anhaltenden lagerungsabhängigen Nystagmus (> 60 s Dauer) ist von einem Anhaften der Otokonien an der Ampulle auszugehen („Cupulolithiasis“).

### Ohruntersuchung

Eine Otoskopie ist bei allen Patient:innen mit Schwindel obligat, dabei steht die Suche nach Entzündungszeichen (z. B. Otorrhö, Rötung/Vorwölbung des Trommelfells, Bläschen/Krusten), Traumata (z. B. Stufenbildung/Blutung im Gehörgang, Trommelfellperforation) und Raumforderungen (z. B. Hornschuppen/epitympanale Retraktion des Trommelfells bei Cholesteatom) im Vordergrund. Bei berichtetem Hörverlust sollte eine orientierende Hörprüfung mittels Stimmgabel (440 Hz; Rinne- und Weber-Test) erfolgen, um zwischen einer Schallleitungsschwerhörigkeit (Rinne negativ auf der betroffenen Seite, Weber lateralisiert zur betroffenen Seite) und einer sensorineuralen Schwerhörigkeit (Rinne positiv, Weber lateralisiert zur gesunden Seite) unterscheiden zu können. Patient:innen mit Auffälligkeiten in der Otoskopie und/oder einem neuartigen Hörverlust sollten HNO-fachärztlich abgeklärt werden.

#### Empfehlung 4.

Die HINTS(plus)-Tests sind *nur* bei Patient:innen mit AVS (d. h. mit Spontan- oder Blickrichtungsnystagmus und persistierender Schwindelsymptomatik) diagnostisch trennscharf zwischen peripheren und zentralen Ursachen. Liegt kein Nystagmus vor (AIS), so sollte primär die graduierte Prüfung der Stand‑/Gangfunktion erfolgen.

#### Empfehlung 5.

Lagerungsmanöver sind insbesondere, aber nicht ausschließlich bei Patient:innen mit intermittierendem, positionsabhängigem Schwindel zu empfehlen. Dabei sollten die posterioren und lateralen Bogengänge geprüft werden.

#### Empfehlung 6.

Es sollte eine neurologische Untersuchung sowie eine Otoskopie und orientierende Prüfung des Gehörs erfolgen.

## Therapieprinzipien

Bei der Behandlung von Schwindel bieten sich pharmakologische, physiotherapeutische, psychotherapeutische/verhaltensneurologische und seltener operative Ansätze an. Oft werden verschiedene Modalitäten kombiniert. Eine an der Pathophysiologie und Ursache orientierte Therapie ist zu priorisieren. Für die wichtigsten Krankheitsbilder sind in Tab. 3 Therapievorschläge zusammengefasst. Dabei ist zu betonen, dass es für viele pharmakologische Therapieansätze nur eine geringe Evidenz gibt. Im Patientengespräch sollte die Prognose der bestehenden Erkrankung thematisiert und eine realistische Erwartung zur Wirklatenz einer therapeutischen Intervention vermittelt werden.

**Tab. 3:** Behandlungsoptionen für die häufigsten neurootologischen Ursachen von Schwindel und/oder Gangstörung

	Medikamentöse Therapien		Nichtmedikamentöse Therapien			
Diagnose	Akutphase/symptomatisch	Prophylaxe	Akutphase/symptomatisch	Prophylaxe/weitere therapeutische Maßnahmen	Follow-up	Empfehlung für weitere Diagnostik/Therapie
*Benigner paroxysmaler Lagerungsschwindel (BPLS)*	Ggf. Antiemetika oder Anxiolytika als Prämedikation vor Repositionsmanövern	Vitamin-D3-Supplementation empfohlen (bei Rezidiv) [26]	Repositionsmanöver (Praxis/Notfall, zuhause, Abb. 3, S1–2), Physiotherapie (inkl. Heimübungen)	Anleitung zur selbstständigen Durchführung von Repositionsmanövern mit Hand-outs, Videos (z. B. https://novel.utah.edu), Smartphone-Apps	Nach 3–7 Tagen (telefonisch, remote per Video, in der Praxis)	Fachärztlich-neurologische und HNO-ärztliche spezialisierte vestibuläre Diagnostik/cMRT bei therapierefraktärer Symptomatik
*Akute unilaterale Vestibulopathie (AUVP)*	Steroide unter Berücksichtigung von Begleiterkrankungen erwägen gemäß S2k-Leitlinie „Vestibuläre Funktionsstörungen“ (DGHNO-KHC/DGN) [27]	Keine	Physiotherapie (vestibuläre Rehabilitationstherapie, VRT) [30]	Anleitung zur selbständigen Durchführung von VRT mit Hand-outs, Videos, Smartphone Apps [31]	Verlaufskontrolle nach 2–4 Wochen empfohlen	Dringliche Zuweisung in die Notfallabteilung, zum HNO-Arzt oder zum Neurologen bei ausgeprägter Klinik oder Verdacht auf zentrale Ursache sowie zur erweiterten qualitativen/quantitativen Vestibularisdiagnostik (siehe Tab. 1 „red flags“)
	Ggf. Antiemetika (Domperidon, Metoclopramid, Ondansetron) und/oder Antivertiginosa (Cinnarizin ± Dimenhydrinat) für max. 2–3 Tage, ggf. raffinierter Trockenextrakt aus Ginkgo-biloba-Blättern (EGb 761) zur Förderung der zentralen Adaptation [28, 29]					
*Ischämischer/hämorrhagischer Schlaganfall („Stroke“)*	Ggf. Antiemetika (Domperidon, Metoclopramid, Ondansetron) zum Transfer	Sekundärprophylaxe gemäß Leitlinien (DGN [32], AHA/ASA [33])	Neurorehabilitation (ambulant, stationär)	Lifestyle-Modifikationen (gemäß Leitlinien zur Sekundärprophylaxe [33])	Regelmäßige Nachkontrollen gemäß Spezialist:in	Umgehende Zuweisung zur nächstgelegenen Stroke Unit mittels Notarzt
				Weitere Maßnahmen siehe S2k-Leitlinie (DGN), Leitlinie AHA/ASA [33]		
*Vestibuläre Migräne (VM)*	Akutmedikation gemäß Guidelines (siehe Migräneattackenbehandlung, Schweizerische Kopfwehgesellschaft, www.headache.ch, und DGN [34])	Entscheid zur Prophylaxe in RS mit Spezialist:in empfohlen	Keine	Lifestyle-Modifikationen	Sofern Beginn einer Langzeitprophylaxe erfolgt: Nachkontrolle nach 10–12 Wochen empfohlen	Zuweisung zum Neurootologen/in ein Schwindelzentrum empfohlen, ggf. parallel cMRT zum Ausschluss sekundärer Schwindel‑/Kopfschmerzursachen
		(siehe Migräne-Langzeitprophylaxe www.headache.ch und DGN [34])		Weitere Maßnahmen siehe Leitlinien DGN und Schweizerische Kopfwehgesellschaft		
*M. Menière*	Antiemetika (Domperidon, Metoclopramid, Ondansetron)	Entscheid zur Prophylaxe in RS mit Spezialist:in empfohlen. Details siehe [27, 35]	Keine	Individuelles multimodales Therapiekonzept, „shared decision making“ mit u. a. folgenden Elementen: Lifestyle-Modifikationen, Hörrehabilitation, Physio‑/Psychotherapie, Selbsthilfegruppen	I. d. R. spezialärztlich	Zuweisung zum Neurootologen/in ein Schwindelzentrum empfohlen, Ausschluss struktureller Ursachen (cMRT), ggf. Hydrops-MRT
				Details siehe [27, 35]		
*Vestibuläre Paroxysmie*	Keine (Attacken i. d. R. zu kurz für symptomatische Therapie)	Entscheidung zur Prophylaxe in RS mit Spezialist:in empfohlen (Carbamazepin, Oxcarbazepin, Lacosamid als Off-Label-Verwendung) [27]	Keine	Keine	I. d. R. spezialärztlich	Zuweisung zum Neurootologen/in ein Schwindelzentrum empfohlen, Ausschluss struktureller Ursachen (cMRT)
„*Persistent postural perceptual dizziness*“* (PPPD)/funktioneller Schwindel*	Verwendung von Antiemetika oder Antivertiginosa nicht empfohlen	Entscheidung zur Prophylaxe in RS mit Spezialist:in empfohlen (i. d. R. Antidepressiva)	Keine	Meist multimodaler Therapieansatz inkl. Patientenedukation (www.vestibular.org), Verhaltenstherapie, Physiotherapie (inkl. Heimübungen) [25]	I. d. R. spezialärztlich	Zuweisung zum Neurootologen/in ein Schwindelzentrum empfohlen, Behandlung der auslösenden Erkrankung (z. B. BPLS, VM)
*Bilaterale Vestibulopathie (BVP)*	Keine	Keine	Keine	Physiotherapie (inkl. Heimübungen mit Hand-outs, Smartphone Apps) Vermeiden ototoxischer Medikamente	Spezialärztliche Verlaufskontrolle nach 6 Monaten empfohlen	Zuweisung zum Neurootologen/in ein Schwindelzentrum empfohlen, Ausschluss zusätzlicher Pathologien (z. B. CANVAS)
				Therapie anderer Sinneseinschränkungen (z. B. Sehen, Hören)		

*AHA* American Heart Association, *ASA* American Stroke Association, *CANVAS* „cerebellar ataxia, neuropathy and vestibular areflexia syndrome“, *cMRT* kranielle Magnetresonanztomographie, *DGHNO-KHC* Deutsche Gesellschaft für Hals-Nasen-Ohren-Heilkunde, Kopf- und Halschirurgie, *DGN* Deutsche Gesellschaft für Neurologie, *RS* Rücksprache, *VRT* vestibuläre Rehabilitationstherapie

## Wichtigste Schwindelsyndrome

### Akutes vestibuläres/akutes Imbalance-Syndrom

Beim AVS/AIS steht die Suche nach einem ischämischen Schlaganfall im Vordergrund (ca. 80 % aller zentralen AVS; Tab. 1, 2 und 3; Abb. 2). Weitere wichtige zentrale Ursachen stellen Medikamentenintoxikationen (z. B. Neuroleptika, Antiepileptika), eine erste Episode einer vestibulären Migräne oder ein akuter Thiaminmangel dar. Davon abzugrenzen ist die akute unilaterale Vestibulopathie (AUVP, „vestibuläre Neuritis“), welche die häufigste Ursache eines peripheren AVS ausmacht. Periphere HINTS(plus)-Befunde sind hierfür suggestiv und sollten eine entsprechende Akutbehandlung (Tab. 3) mit sich führen. Dabei ist auf eine Limitierung der Einnahmedauer von Antivertiginosa und Antiemetika auf max. 2–3 Tage zu achten, da ansonsten die zentrale Kompensation negativ beeinflusst wird.

### Episodisches vestibuläres Syndrom

Beim EVS ist die Unterscheidung getriggert vs. spontan essenziell (Abb. 2). Ist in den Lagerungsmanövern bei Patient:innen mit getriggertem EVS (t-EVS) ein BPLS-typischer Nystagmus nachweisbar, so sollte das passende Repositionsmanöver erfolgen (Abb. 3). Lässt sich kein Nystagmus auslösen oder tritt Schwindel nur beim Aufsitzen auf, so sollte eine orthostatische Hypotension gesucht werden, wobei das Fehlen des Auslösens eines Nystagmus bei der Lagerung einen bereits abgeklungenen BPLS nicht ausschließt.

Zur Behandlung des BPLS des posterioren BG (ca. 80 %) hat sich das Epley-Manöver (Abb. 3c) oder alternativ das Semont-plus-Manöver (Abb. S1) etabliert [16, 18]. Liegt ein BPLS des lateralen BG vor (ca. 20 %), so sollte bevorzugt ein Gufoni-Manöver (Abb. 3d) erfolgen, alternativ ein 360°-Barbecue-Manöver über das nichtbetroffene Ohr mit anschließendem Ruhen auf dem nichtbetroffenen Ohr („roll and rest“; Abb. S2; [19]). Liegt eine geotrope Variante vor, so erfolgt das Gufoni-Manöver „nose down“ auf die nichtbetroffene Seite, liegt die apogeotrope Variante vor, so erfolgt es „nose up“ auf die betroffene Seite. Die Lagerung erfolgt beim Gufoni-Manöver immer auf die Seite mit weniger Nystagmus. Lässt sich dies im Supine-Roll-Manöver nicht eindeutig festlegen, so sollte die Patient:innen nach der Intensität der Schwindelbeschwerden befragt und das Gufoni-Repositionsmanöver auf der Seite mit weniger Schwindel begonnen werden.

Nach dem Repositionsmanöver sollte ein Lagerungsmanöver zur Erfolgskontrolle (und ggf. erneuter Reposition) erfolgen. Selbstrepositionen sind bei Patient:innen mit rezidivierendem BPLS sinnvoll. Eine Nachkontrolle (ggf. „remote“) ist empfohlen [20]. Patient:innen sollten über nachfolgende, unspezifische Beschwerden (Gangunsicherheit, Benommenheitsgefühl) für einige Tage informiert und auf das Rezidivrisiko von 15–20 % über 12 Monate aufmerksam gemacht werden. Ist aufgrund von Immobilität, Wirbelsäulenpathologien oder eines (extra)pyramidalmotorischen Syndroms die Durchführung von Manövern auf der Liege nicht möglich, so bietet sich die Testung/Behandlung auf einem manuellen Drehstuhl an (z. B. TRV Chair, Fa. Interacoustics, Dänemark, oder Rotundum, Fa. Balcare, Küsnacht, Schweiz). Sind Repositionsmanöver bei vermutetem BPLS wiederholt erfolglos, ist das Nystagmusmuster atypisch oder finden sich fokal-neurologische Auffälligkeiten, so stellt dies eine „red flag“ dar (Tab. 1; Abb. 2).

Beim spontanen EVS (s-EVS) steht die Differenzierung vestibuläre Migräne (VM) vs. M. Menière im Vordergrund (Abb. 2). Während bei der VM eine positive Anamnese für Migränekopfschmerzen zwingend vorliegen muss, ist eine reintonaudiometrisch dokumentierte sensorineurale Tieftonschwerhörigkeit zur Diagnose des „sicheren“ M. Menière Pflicht. Die diagnostischen Kriterien der VM verlangen mindestens 5 Schwindelepisoden moderater/hoher Intensität (Symptomdauer 5 min–72 h) bei gleichzeitigem Vorliegen von Migränesymptomen in mindestens jeder zweiten Attacke sowie die Diagnose einer Migräne [21] (für therapeutische Maßnahmen siehe [22] sowie Tab. 3). Für die Diagnose eines M. Menière sind ≥2 Episoden mit vestibulärer und cochleärer Symptomatik (Dauer 20 min–12 h) erforderlich [23]. Einige therapeutische Optionen beim M. Menière sind in Tab. 3 abgebildet und werden von HNO-Spezialist:innen individuell angepasst.

### Chronisches vestibuläres Syndrom

Bei persistierender Symptomatik gilt es, aggravierende und provozierende Faktoren zu identifizieren. Treten Schwindel oder Gleichgewichtsstörungen nur im Stehen/Gehen auf, ist nach einer PNP, einem chronischen einseitigen Vestibularisausfall und einer BVP oder einer zentralen (neurodegenerativen, chronisch-zerebrovaskulären) Ursache zu suchen. Begleitendes Verschwommensehen bei raschen Kopfdrehungen/beim Gehen spricht für eine BVP. Ebenso weisen das Vorliegen eines beidseitig abnormen KIT, einer Sturzneigung im Romberg-Test auf der Schaummatte sowie einer Abnahme des Visus um ≥ 2 Linien bei Prüfung während Kopfschütteln (sog. dynamische Sehschärfe) auf eine BVP hin (siehe [24]). Lässt sich ein chronischer Schwindel nicht durch eine Störung sensorischer Funktionen erklären, sollte insbesondere auch auf eine zentrale oder funktionelle Ursache geachtet werden. Bei V. a. zentrale Ursache (z. B. Kleinhirnzeichen, Hypokinesie) ist die fachärztlich-neurologische Vorstellung obligat.

Besteht ein anhaltender Schwankschwindel über ≥3 Monate mit Exazerbation beim Aufstehen, aktiver oder passiver Bewegung sowie beim Betrachten bewegter/komplexer visueller Stimuli, welcher zu einer relevanten Beeinträchtigung im Alltag führt, spricht dies für einen funktionellen Schwindel („persistent postural perceptual dizziness“, PPPD). Häufig liegt als Auslösefaktor ein Schwindelereignis oder eine psychosoziale Belastungssituation vor [25]. Die Therapie ist multimodal (Tab. 3).

#### Empfehlung 7.

Beim BPLS sollten Repositionsmanöver (Semont plus/Epley für den posterioren BG; Gufoni/Barbecue für den lateralen BG) durchgeführt werden.

#### Empfehlung 8.

Bei Verdacht auf Schlaganfall ist die notfallmäßige Zuweisung zur nächstgelegenen Stroke Unit essenziell, bei einem akuten Thiaminmangel die hochdosierte B1-Supplementation vordringlich.

#### Empfehlung 9.

Antivertiginosa/Antiemetika sollten auf eine Therapiedauer von max. 2–3 Tage begrenzt werden.

#### Empfehlung 10.

Die Behandlung der vestibulären Migräne, des M. Menière und auch des funktionellen Schwindels sollte in spezialärztlicher Absprache erfolgen.

## Ausblick

Angesichts bestehender Wissenslücken von Primärversorger:innen in der Betreuung von Patient:innen mit Schwindel [1] ist die Weiter- und Fortbildung essenziell, um die Rate an unklaren Fällen, spezialärztlichen Zuweisungen wie auch nicht zielgerichteter Zusatzdiagnostik zu senken und gleichzeitig die Prognose zu verbessern. Primärversorger:innen sollten in der Anwendung der HINTS-Tests und der Lagerungs‑/Repositionsmanöver bei BPLS geschult werden [2].

## Fazit für die Praxis

- Bei Schwindel ist eine strukturierte Vorgehensweise inkl. gezieltem Erfragen der Dauer und Häufigkeit der Episoden und Provokationsfaktoren sowie eine fokussierte neurootologische Untersuchung einschließlich der Suche nach subtilen okulomotorischen Zeichen essenziell („TiTrATE“-Herangehensweise).
- Die Identifikation gefährlicher Ursachen ist prioritär, beim akuten vestibulären Syndrom und akuten Imbalance-Syndrom sind dies v. a. vertebrobasiläre Ischämien und beim episodischen vestibulären Syndrom kardiale Arrhythmien und eine TIA.
- Primärversorger:innen sollten in der praktischen Anwendung der HINTS sowie der Lagerungs- und Repositionsmanöver (insbesondere für den lateralen BG) geschult werden.
- Medikamentöse Therapien bei Schwindel sollten möglichst evidenzbasiert durchgeführt werden und periodisch bezüglich Wirksamkeit und Verträglichkeit reevaluiert werden.

## Supplementary Information

ESM: Tab. S1: Wichtigste Begriffe – Definitionen; Abb. S1: Das Semont-plus-Manöver zur Behandlung des BPLS des posterioren Bogengangs; Abb. S2: Das 360° Barbecue-Manöver („Lempert-Manöver“) zur Behandlung des BPLS des lateralen Bogengangs (hier exemplarisch für den rechten lateralen Bogengang, geotrope Variante)

## Abkürzungen

AIS: Akutes Imbalance-Syndrom
AUVP: Akute unilaterale Vestibulopathie
AVS: Akutes vestibuläres Syndrom
BG: Bogengang
BPLS: Benigner paroxysmaler Lagerungsschwindel
BRN: Blickrichtungsnystagmus
BVP: Bilaterale Vestibulopathie
CT: Computertomographie
CVS: Chronisches vestibuläres Syndrom
EVS: Episodisches vestibuläres Syndrom
HINTS: „Head impulse, nystagmus, test of skew“
KIT: Kopfimpulstest
MRT: Magnetresonanztomographie
PNP: Polyneuropathie
PPPD: „Persistent postural perceptual dizziness“
s: Spontan
SN: Spontannystagmus
t: Getriggert
TIA: Transient-ischämische Attacke
TiTrATE: „Timing, Triggers And Targeted Examinations“
VM: Vestibuläre Migräne
VOR: Vestibulookulärer Reflex
