# Erratum zu: Diagnostik und Therapie bei Schwindel

**DOI:** 10.1007/s00106-025-01669-2

**Published:** 2025-09-22

**Authors:** Alexander Andrea Tarnutzer, Hassen Kerkeni, Suzie Diener, Roger Kalla, Claudia Candreia, Renato Piantanida, Raphaël Maire, Antje Welge-Lüssen, Joris Budweg, Andreas Zwergal, Julia Dlugaiczyk

**Affiliations:** 1 Neurologie, Kantonsspital Baden, Baden, Schweiz; 2 Universität Zürich (UZH), Zürich, Schweiz; 3 Klinik für Neurologie, Inselspital Bern, Universität Bern, Bern, Schweiz; 4 Praxis „Neurologie St. Gallen“, St. Gallen, Schweiz; 5 Klinik für Hals‑, Nasen‑, Ohren- und Gesichtschirurgie, Kantonsspital Luzern, Luzern, Schweiz; 6 Servizio di Otorinolaringoiatria, Ente Ospedaliero Cantonale, Lugano, Schweiz; 7 Service d’oto-rhino-laryngologie, Centre hospitalier universitaire vaudois, Lausanne, Schweiz; 8 Hals-Nasen-Ohren-Klinik, Universitätsspital Basel, Basel, Schweiz; 9 Hausarztzentrum Bethesda, Basel, Schweiz; 10 Neurologische Klinik und Poliklinik, LMU Klinikum, München, Deutschland; 11 Deutsches Schwindel- und Gleichgewichtszentrum (DSGZ), LMU Klinikum, München, Deutschland; 12 Klinik für Ohren‑, Nasen‑, Hals- und Gesichtschirurgie, Universitätsspital Zürich (USZ), Rämistrasse 100, 8091 Zürich, Schweiz; 13 Interdisziplinäres Zentrum für Schwindel und neurologische Sehstörungen, Universitätsspital Zürich, Zürich, Schweiz

**Erratum zu:**

**HNO 2025**

10.1007/s00106-025-01598-0

In der Tab. 3 zu diesem Artikel war der Eintrag in der zweiten Spalte unter der Überschrift „Medikamentöse Therapien/Akutphase/symptomatisch“ nicht korrekt.

Er hätte richtigerweise „Ggf. Antiemetika (Domperidon, Metoclopramid, Ondansetron) und/oder Antivertiginosa (Cinnarizin ± Dimenhydrinat) für max. 2–3 Tage, ggf. raffinierter Trockenextrakt aus Ginkgo-biloba-Blättern (EGb 761) zur Förderung der zentralen Adaptation [28, 29]“ lauten müssen, anstelle von „Ggf. Antiemetika (Domperidon, Metoclopramid, Ondansetron) und/oder Antivertiginosa (Cinnarizin ± Dimenhydrinat) für max. 2–3 Tage, ggf. raffinierter Trockenextrakt aus Ginkgo-biloba-Blättern (EGb 716) zur Förderung der zentralen Adaptation [28, 29]“.

Der Originalbeitrag wurde korrigiert.

