# Supplementary material for: Diagnosis and treatment of vertigo and dizziness: Interdisciplinary guidance paper for clinical practice. German version
Source: HNO. 2025 Apr 16;73(7):461–73. [Article in German] doi: 10.1007/s00106-025-01598-0 (PMC12185573; doi:10.1007/s00106-025-01598-0)
Supplement: Supplementary file 1 — ESM: Tab. S1: Wichtigste Begriffe – Definitionen; Abb. S1: Das Semont-plus-Manöver zur Behandlung des BPLS des posterioren Bogengangs; Abb. S2: Das 360° Barbecue-Manöver („Lempert-Manöver“) zur Behandlung des BPLS des lateralen Bogengangs (hier exemplarisch für den rechten lateralen Bogengang, geotrope Variante) [file 106_2025_1598_MOESM1_ESM.pdf]

Supplement

Tabelle S1: Wichtigste Begriffe – Definitionen (1, 2)

**Begriff**	**Definition**
**Symptome**	
Dizziness	Wahrnehmung einer gestörten oder verminderten räumlichen Orientierung ohne Vorliegen einer illusionären oder verzerrten Eigenbewegung.
Vertigo	Wahrnehmung einer Eigenbewegung, ohne dass tatsächlich eine Eigenbewegung besteht oder die Wahrnehmung einer verzerrten Eigenbewegung während einer regulären Kopfbewegung
**Schwindelsyndrome**	
Akutes vestibuläres Syndrom (AVS)	Beschwerdebild mit akutem prolongiertem (>24h anhaltendem) Schwindel mit begleitend Nausea/Vomitus, Gangunsicherheit, Bewegungsintoleranz sowie einem Spontan- oder Blickrichtungsnystagmus.
Akutes Imbalance Syndrom (AIS)	Beschwerdebild mit akuter prolongierter Stand- und Gangunsicherheit (und ggf. Schwindel) ohne Spontan- oder Blickrichtungsnystagmus.
Episodisches vestibuläres Syndrom (EVS)	Beschwerdebild mit intermittierendem Schwindel von einer Dauer von Sekunden, Minuten, Stunden oder auch Tagen und symptomfreien Intervallen zwischen den Episoden. Auftreten spontan oder ausgelöst durch z.B. Positionsänderungen
Chronisches vestibuläres Syndrom (CVS)	Beschwerdebild mit chronischem (persistierendem) Dreh- oder Schwankschwindel
**Klinische Befunde**	
Spontannystagmus	Unwillkürliche rhythmische Augenbewegungen in Primärposition (Blick geradeaus), oftmals mit einer langsamen und einer schnellen (die Schlagrichtung des Nystagmus definierenden) Phase.
Blickrichtungsnystagmus	Nystagmus, welcher seine Schlagrichtung in Abhängigkeit der Blickrichtung umkehrt und nur in exzentrischer Blickposition (horizontal oder vertikal) auftritt. Zugrunde liegt eine eingeschränkte Fähigkeit, eine exzentrische Blickposition stabil zu halten, und es tritt ein Drift (langsame Phase) der Augen zur Primärposition hin (zentripetal) auf, gefolgt von einer kompensatorischen (schnellen Phase) Korrekturbewegung der Augen nach aussen (zentrifugal).
Skew deviation	Vertikale Divergenz der Sehachse durch Läsionen entlang der peripheren oder zentralen vestibulären Bahnen, welche mittels alternierendem Abdecktest (Test of Skew) geprüft wird.
Vestibulo-okulärer Reflex (VOR)	Reflexbogen, welcher die Fixation eines Objektes im Raum mit den Augen während Kopfbewegungen ermöglicht und bei Durchführung des Kopfimpulstests untersucht wird. Beim VOR werden Eingangssignale aus den Bogengängen im Innenohr über den Vestibularisnerven zu den Vestibulariskernen im Hirnstamm weitergeleitet und von dort über verschiedene Bahnen zu den Augenmuskelkernen und den Augenmuskeln weitergeleitet. Es erfolgt in der Folge bei Kopfdrehung eine kompensatorische Gegenbewegung der Augen.
HINTS (3)	Prüfung subtiler okulomotorischer Zeichen am Patientenbett mittels dreier klinischer Tests (horizontaler Kopfimpulstest (**H**ead-**I**mpulse), Prüfung der exzentrischen Blickhaltefunktion (**N**ystagmus), vertikale

	Divergenz im alternierenden Abdecktest (**T**est-of-**S**kew)) beim Patienten mit AVS. Als «zentral» gelten die HINTS, sofern eines oder mehrere der folgenden Zeichen vorliegen: normaler Kopfimpulstest, Vorliegen eines Blickrichtungsnystagmus, Vorliegen einer Skew deviation. Als «peripher» gelten die HINTS, sofern ein abnormer Kopfimpulstests vorliegt **und** kein Nachweis eines Blickrichtungsnystagmus oder einer Skew deviation vorliegt.
HINTS plus (4)	Prüfung der HINTS (siehe oben) sowie zusätzliche Suche nach einer neu aufgetretenen, einseitigen Hörminderung bei Patient:innen mit peripheren HINTS. Die HINTS plus gelten als «zentral», sofern eine neu aufgetretene, einseitige Hörminderung nachweisbar ist (Prüfung mittels Flüsterzahlen, Fingerreiben, Stimmgabel).

Abbildung S1 – Das Semont-plus Manöver zur Behandlung des BPLS des posterioren Bogenganges

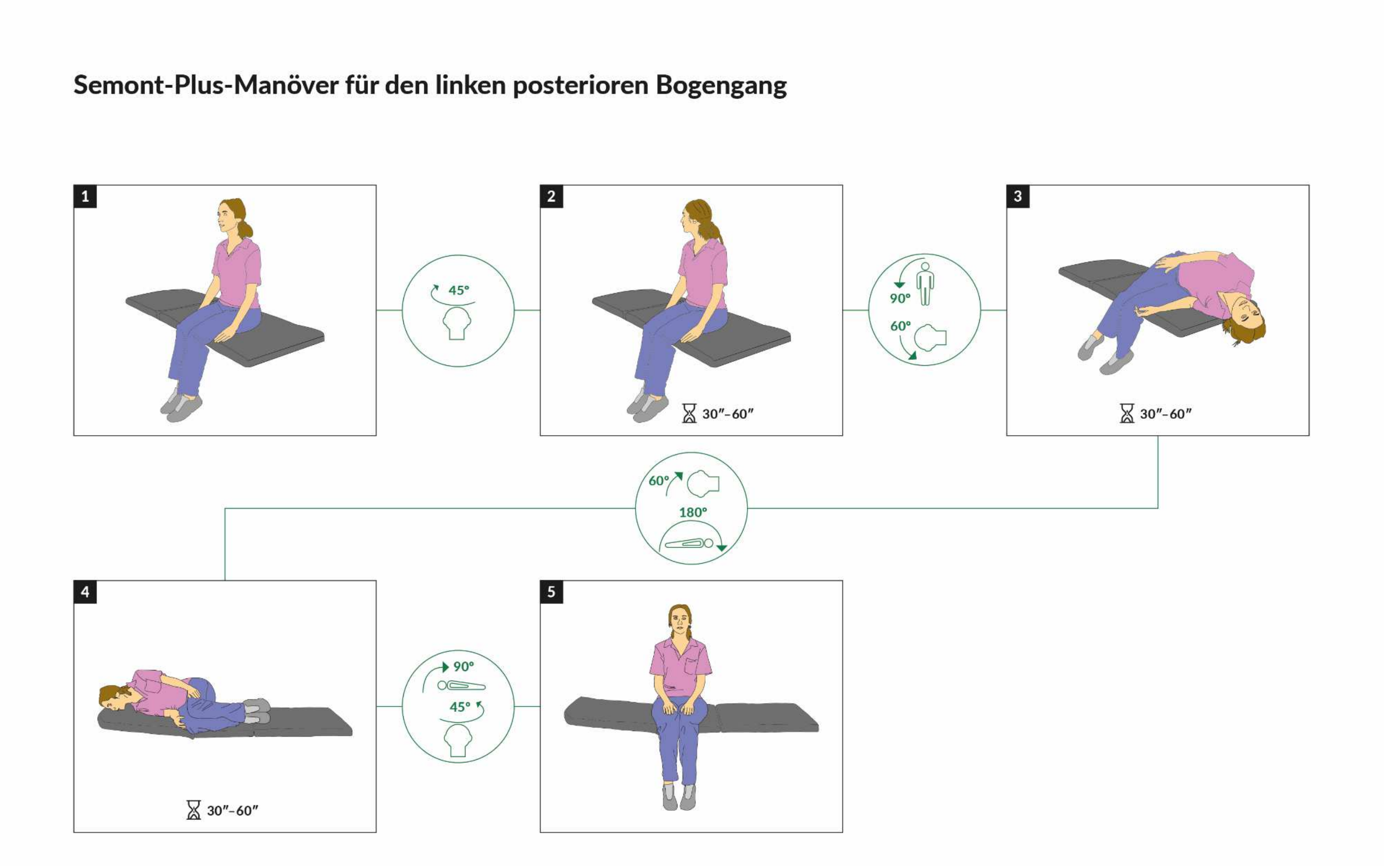

Abbildung S2 – Das 360° Barbecue-Manöver («Lempert Manöver») zur Behandlung des BPLS des lateralen Bogenganges (hier exemplarisch für den rechts-lateralen Bogengang (geotrope Variante))

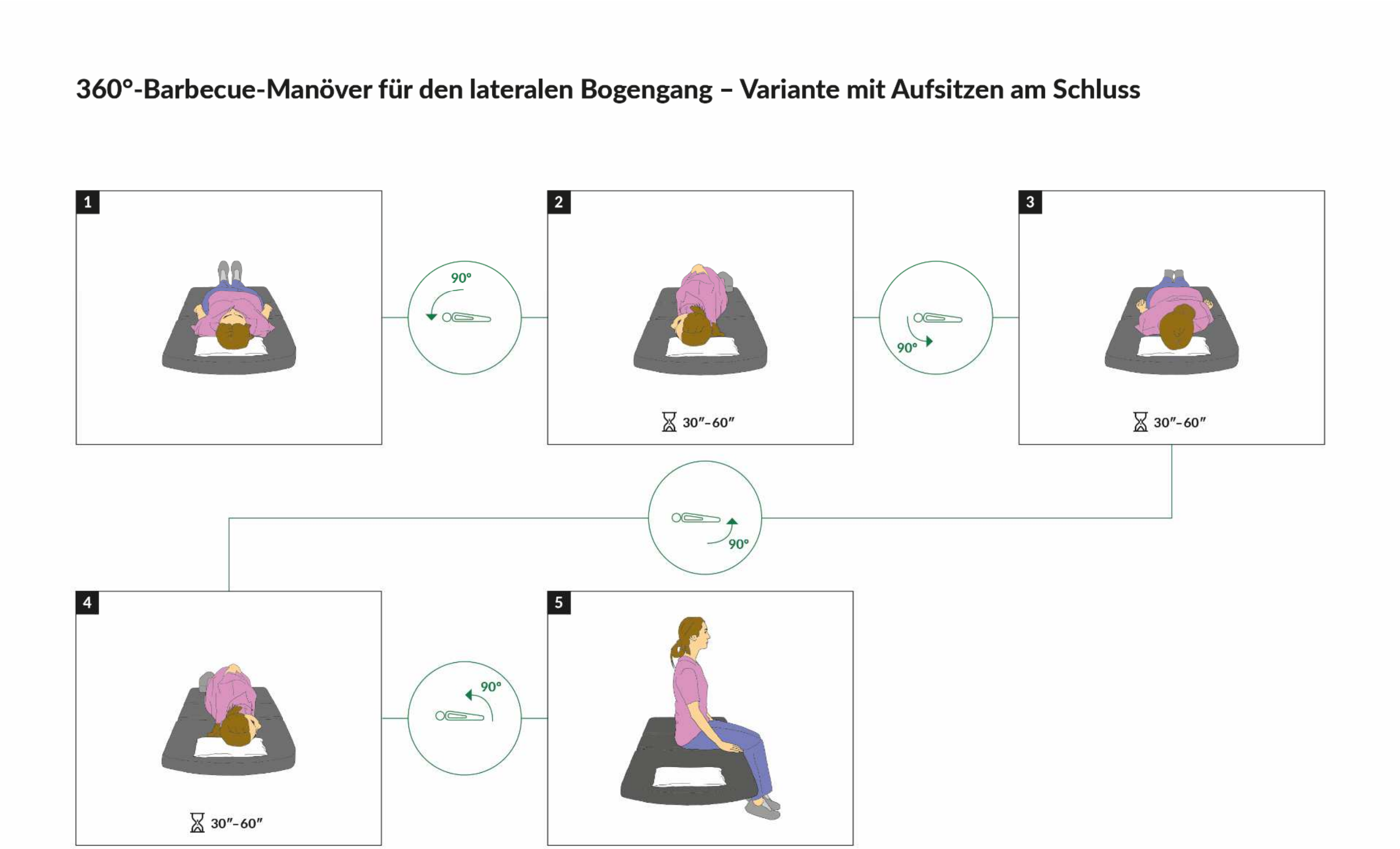

Legende: für die geotrope Variante erfolgt die Lagerung auf diejenige Seite mit weniger ausgeprägtem Nystagmus, für die apogeotrope Variante (hier nicht abgebildet) erfolgt die Lagerung auf diejenige Seite mit dem stärkeren Nystagmus.

Referenzen

1. Newman-Toker DE, Edlow JA. TiTrATE: A Novel, Evidence-Based Approach to Diagnosing Acute Dizziness and Vertigo. Neurol Clin. 2015;33(3):577-99, viii.
2. Bisdorff AR, Staab JP, Newman-Toker DE. Overview of the International Classification of Vestibular Disorders. Neurol Clin. 2015;33(3):541-50, vii.
3. Kattah JC, Talkad AV, Wang DZ, Hsieh YH, Newman-Toker DE. HINTS to diagnose stroke in the acute vestibular syndrome: three-step bedside oculomotor examination more sensitive than early MRI diffusion-weighted imaging. Stroke. 2009;40(11):3504-10.
4. Newman-Toker DE, Kerber KA, Hsieh YH, Pula JH, Omron R, Saber Tehrani AS, et al. HINTS outperforms ABCD2 to screen for stroke in acute continuous vertigo and dizziness. Acad Emerg Med. 2013;20(10):986-96.
